# An Unusual Cytomegalovirus Ocular Manifestation in a Non-HIV Patient

**DOI:** 10.7759/cureus.21947

**Published:** 2022-02-05

**Authors:** Emily Ng Ming Choo, Othmaliza Othman, Amelia Lay Suan Lim

**Affiliations:** 1 Ophthalmology, University Kebangsaan Malaysia, Kuala Lumpur, MYS; 2 Ophthalmology, Hospital Kuala Lumpur, Kuala Lumpur, MYS

**Keywords:** cmv pcr, cytomegalovirus retinitis, endotheliitis, ganciclovir, cytomegalovirus

## Abstract

A 60-year-old Aboriginal man with underlying severe exfoliative dermatitis, treated with oral azathioprine and oral prednisolone, presented with left painful red eye for ten days. On initial presentation, left eye vision was poor at hand motion. There was corneal endotheliitis over the left eye with severe anterior chamber inflammation obscuring the fundus view. B-scan ultrasonography showed evidence of vitritis with a flat retina. An urgent aqueous tap for viral polymerase chain reaction (PCR) yielded positive cytomegalovirus (CMV) DNA results. As CMV infection commonly affects immunosuppressed individuals, his systemic immunosuppressants were withheld temporarily. He was successfully treated with combination intravenous ganciclovir, intravitreal ganciclovir 2mg/0.1ml, and topical ganciclovir 2%. His vision improved significantly from hand motion to 20/40. There was no reactivation of CMV infection post-treatment.

## Introduction

Cytomegalovirus (CMV) is a double-stranded DNA virus under the herpes virus family, commonly found dormant in all healthy adult individuals. CMV retinitis usually affects immunocompromised individuals with low CD4 count or white cell count. On the other hand, CMV corneal endotheliitis is commonly seen in immunocompetent individuals. It is usually associated with corneal edema, coin-shaped keratic precipitates (KPs), and elevated intraocular pressure [1-4]. We report a case of concurrent CMV endotheliitis and CMV retinitis in a non-HIV patient.

## Case presentation

A 60-year-old Aboriginal man with underlying hypertension, diabetes mellitus, and exfoliative dermatitis presented with left eye pain, redness, and generalized blurring of vision for ten days. He was afebrile and systemic review for infection was unremarkable. He denied tuberculosis (TB) contact, and there were no symptoms suggestive of TB infection, such as chronic cough, hemoptysis, night sweat, or constitutional symptoms. 

He has been treated with oral immunosuppressants for exfoliative dermatitis for the past one year. He was initially treated with oral prednisolone 0.5mg/kg per day combined with oral methotrexate (MTX) 10mg weekly. However, oral MTX has later switched to oral azathioprine 100mg OD due to poor control of inflammation.

**Figure 1:**
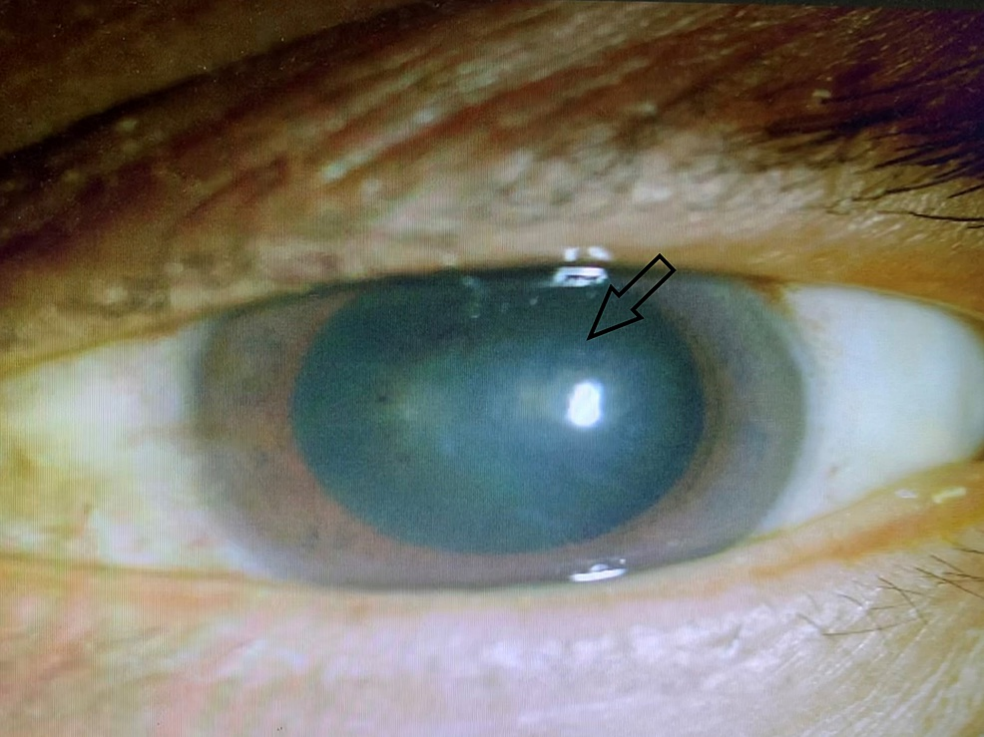
Left eye central disciform corneal endotheliitis (arrow) at initial presentation.

On examination, his visual acuity was 20/30 on the right eye, while left vision was poor at hand motion. Corneal sensation was intact for both eyes. The intraocular pressure was 15mmHg OD and 20mmHg OS. Anterior segment (AS) examination revealed that his conjunctiva was congested over the left eye. There was a central disciform corneal edema with diffuse stellate KPs in the left eye (Figure 1). Left eye anterior chamber (AC) cells were 3+ with no apparent iris atrophy. Right eye AS examination was normal.

**Figure 2:**
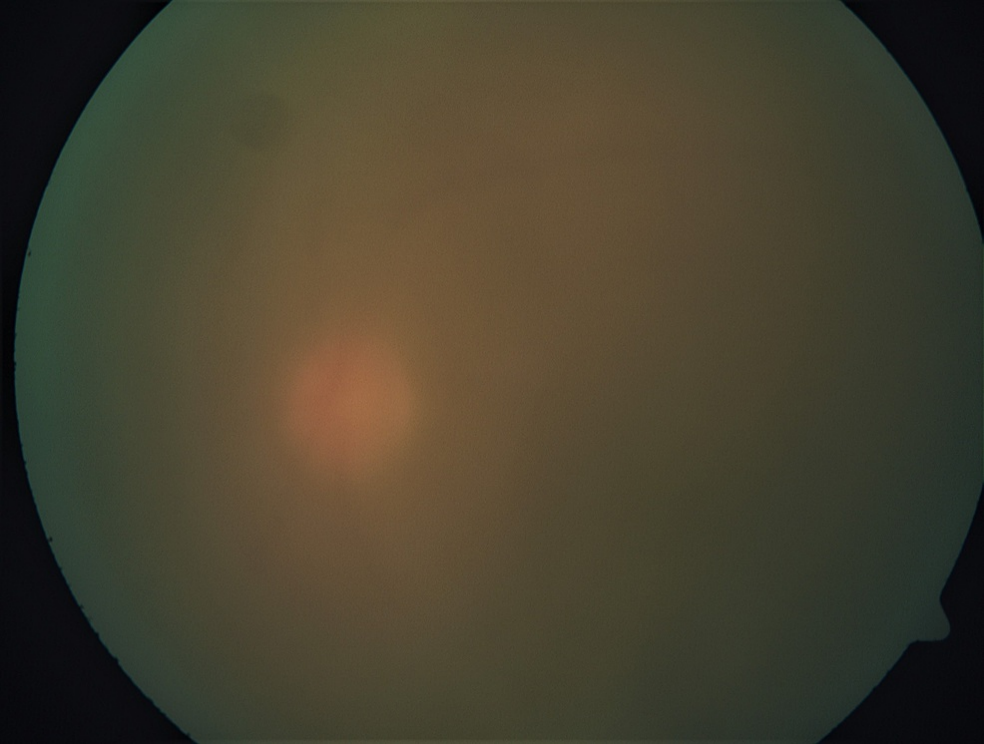
Left eye colour fundus photography with hazy fundus view (dense vitritis) at initial presentation.

Left eye fundus examination showed limited fundus view due to dense vitritis of 3+ (Figure 2). The optic disc was barely visible, and it was hyperemic. B-scan examination showed vitreous opacities; however, the retina was still flat. The right eye fundus showed a normal disc without diabetic retinopathy.

An urgent aqueous tap for viral PCR (herpes simplex virus (HSV), varicella zoster virus (VZV), and CMV) was performed. PCR testing revealed significantly high copies of CMV DNA (3.1 x 106 IU/mL), while PCR tests for HSV and VZV DNA were not detected. The serology CMV IgG was positive as well; however, IgM was negative. His total white cell count was 11.0 x 10^9^ /L with a relatively low lymphocyte count of 1.0 x 10^9^ /L. He was treated for a lung infection at the same time as the gram staining of the sputum sample showed gram-positive cocci. He has been given oral augmentin 625mg twice daily for a week. However, the final sputum culture did not grow any positive culture. 

**Figure 3:**
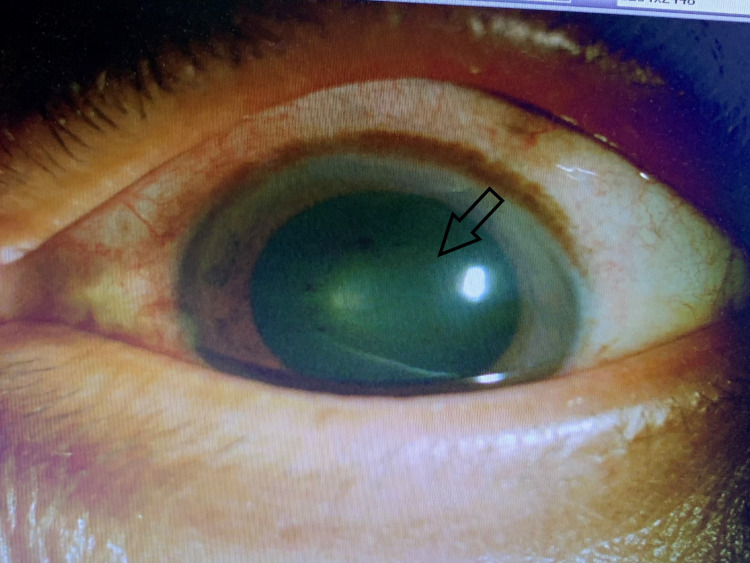
Left eye central disciform corneal endotheliitis has resolved (arrow) after treatment.

**Figure 4:**
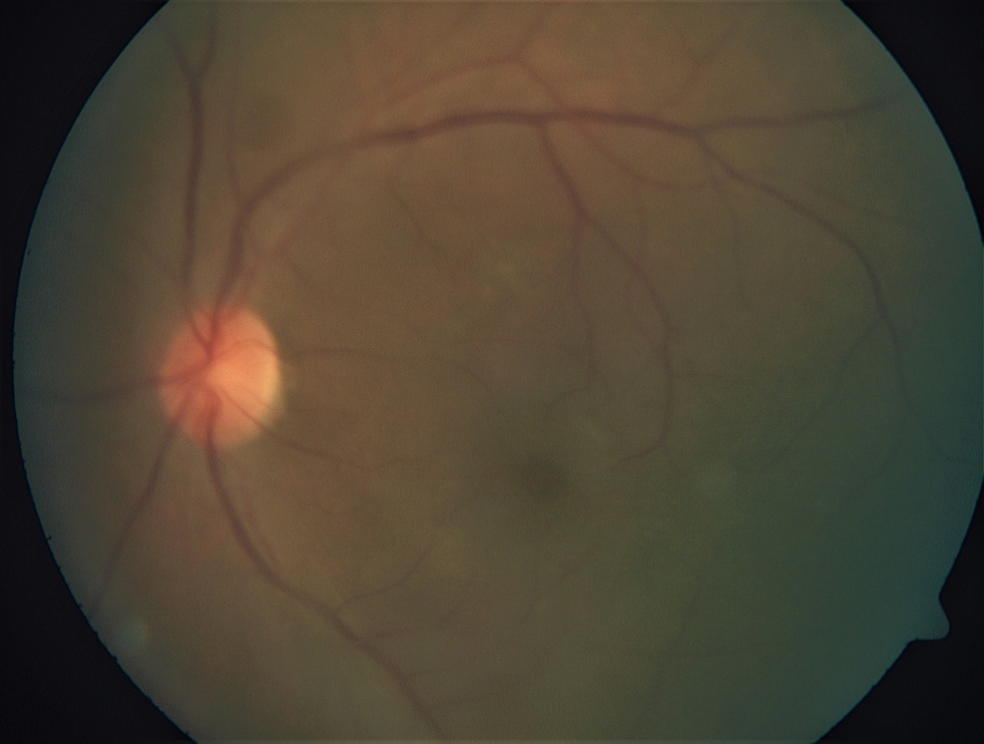
The vitritis of the left eye has significantly improved after intravenous and intravitreal ganciclovir.

**Figure 5:**
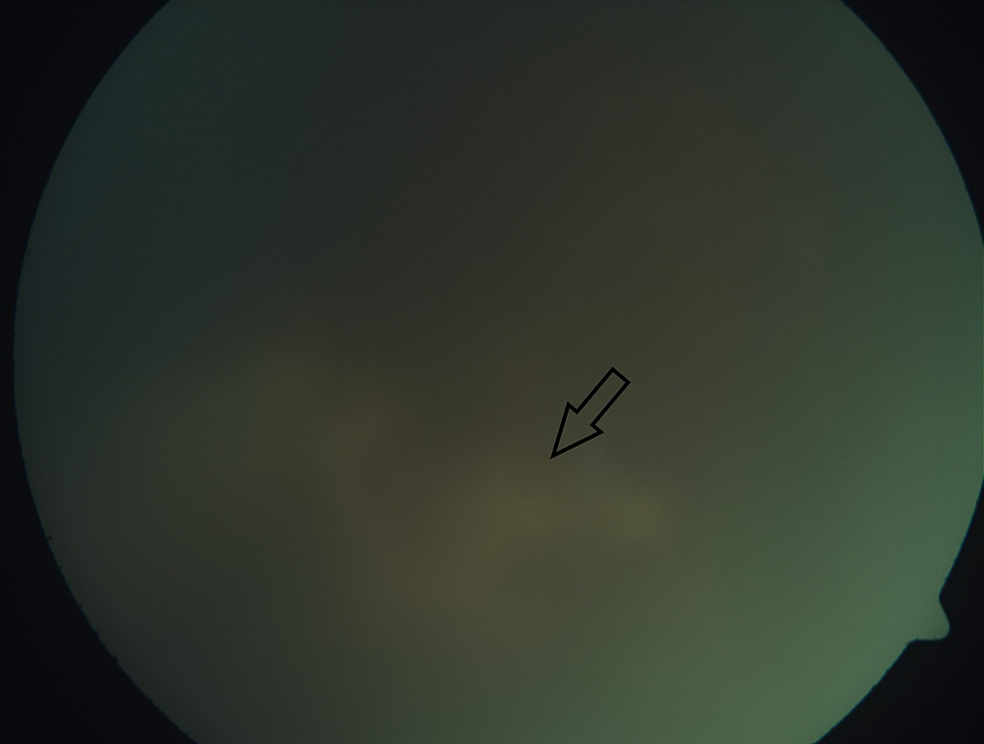
Left eye retinitis lesion (arrow) at the inferior retina from the 6 to 9 o'clock region involving mid to far periphery retina at initial presentation.

Intravenous ganciclovir 5mg/kg twice daily (induction dose) was initiated and administered for 14 days. He was also given left eye intravitreal ganciclovir 2mg/0.1ml biweekly. Besides, topical ganciclovir 2% of five times per day and topical prednisolone 1% were started for AS involvement. There was a tremendous improvement after five days of intravenous ganciclovir and two doses of intravitreal ganciclovir. Left eye disciform endotheliitis resolved significantly, and the left eye fundus view became clearer (Figures 3-4). A large retinitis lesion was seen at the inferior retina at the 6 o'clock region extending up to the 9 o'clock region involving the mid to far periphery retina (Figure 5). Despite severe intraocular inflammation, optical coherence tomography (OCT) of the left eye macula did not show any cystoid macula edema.

**Figure 6:**
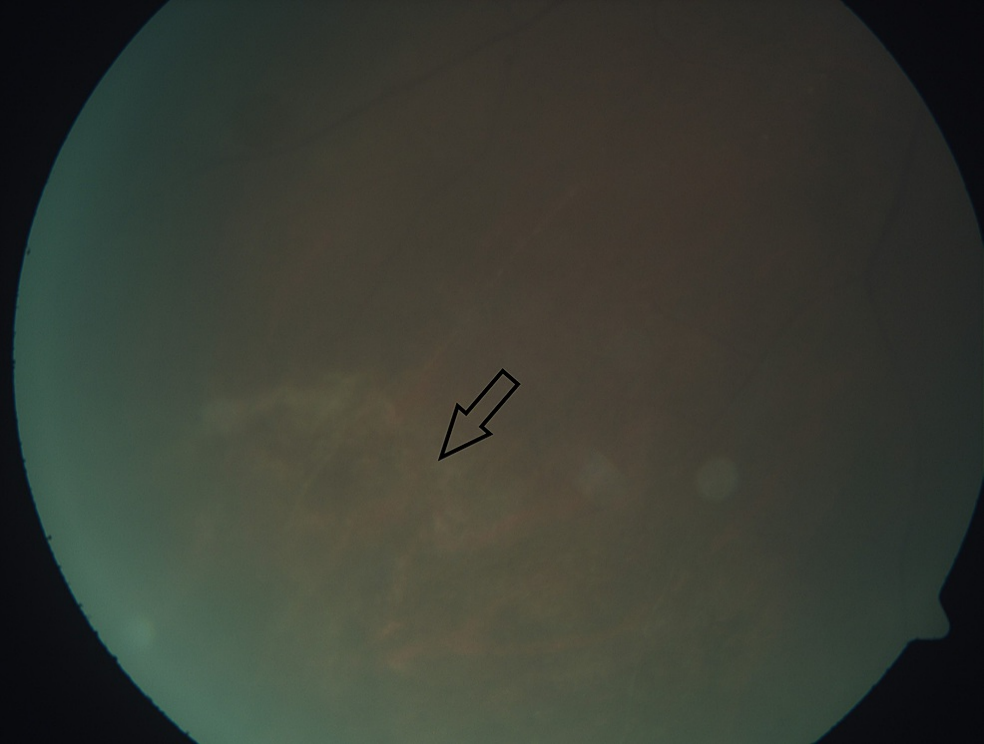
The retinitis lesion has scarred up (arrow) after treatment.

Nonetheless, the patient did not have any spike in intraocular pressure throughout the monitoring. Based on AC activities, he was on topical ganciclovir 2% five times a day with slow tapering of frequency over six months. A total of five doses of intravitreal ganciclovir was given to him. After completing intravenous ganciclovir, the left eye visual acuity improved to 20/40, and the retinitis scarred up (Figure 6). 

## Discussion

CMV retinitis is a severe ocular problem that can lead to blindness. CMV retinitis characteristics and treatment in HIV individuals and individuals on immunosuppressant post organ transplantation have been well studied [5-8]. On the other hand, CMV endotheliitis and hypertensive anterior uveitis usually occur in immunocompetent individuals. Concurrent CMV retinitis and CMV endotheliitis are rare in immunosuppressed individuals. Moreover, our patient presented with dense vitritis. It is unusual to see ocular CMV infection with dense vitritis in immunocompromised patients.

CMV endotheliitis alone can lead to vision loss in the case of corneal edema and corneal decompensation. Delayed diagnosis and treatment can reduce endothelial cell count (ECC), leading to corneal decompensation. If left untreated, CMV reactivation can occur in corneal graft, which can cause the decrease of ECC in the corneal graft and render graft failure [9].

Diagnosis of CMV retinitis in our patient can be challenging as the fundus view was hazy due to dense vitritis and corneal edema. The presence of dense vitritis is an unusual feature of CMV retinitis. CMV retinitis is an opportunistic infection primarily seen in immunocompromised individuals. We postulate that our patient could have developed CMV retinitis at the periphery retina when his immune system was compromised at the beginning of the ocular infection. It was followed by the development of corneal endotheliitis and dense vitritis when immune recovery was reinstituted. The features of corneal endotheliitis are highly suggestive of viral aetiology. The positive yield of CMV DNA in aqueous leads to early diagnosis and successful treatment in our patient. The CMV DNA PCR can be negative in the later stage of the disease, especially with mild ocular inflammation due to low viral load [10]. Therefore, a late diagnostic tap may give rise to false-negative results for CMV infection if the AC flare is minimal.

There is a tendency for reactivation of CMV endotheliitis after stopping treatment. Thus, individuals with corneal endotheliitis or anterior uveitis usually require a longer duration of treatment [11-13]. Due to the severe toxicity of systemic ganciclovir, treatment of CMV endotheliitis with topical ganciclovir has gained popularity. However, up to date, there is no consensus on the optimal treatment regime and duration of topical ganciclovir [14].

Topical ganciclovir and topical prednisolone are used in treating corneal endotheliitis to target both infectious and inflammatory components. Topical ganciclovir 2% was effective and safe for anterior segment inflammation secondary to CMV infection [15-17]. This preparation is beneficial when commercially prepared topical ganciclovir is not widely available. It also shows good corneal penetration, and long-term usage has little systemic side effect [18-19]. Our patient was on topical ganciclovir 2% for six months without any reactivation of CMV infection.

## Conclusions

This case report highlights the possible rare concurrent CMV retinitis and CMV corneal endotheliitis. CMV infection is an important cause of keratouveitis. Hence, a relatively safe and straightforward aqueous tap should be done in all individuals with keratouveitis for early definitive diagnosis and appropriate treatment. Delay in diagnosis can lead to recurrent inflammation and potential blindness.
